# Reduced Expression of Antimicrobial Protein Secretory Leukoprotease Inhibitor and Clusterin in Chronic Rhinosinusitis with Nasal Polyps

**DOI:** 10.1155/2021/1057186

**Published:** 2021-01-07

**Authors:** Yanran Huang, Ming Wang, Yu Hong, Xiangting Bu, Ge Luan, Yang Wang, Ying Li, Hongfei Lou, Chengshuo Wang, Luo Zhang

**Affiliations:** ^1^Department of Otolaryngology, Head and Neck Surgery, Beijing Tongren Hospital, Capital Medical University, Beijing 100730, China; ^2^Beijing Key Laboratory of Nasal Diseases, Beijing Institute of Otolaryngology, Beijing 100005, China; ^3^Department of Allergy, Beijing Tongren Hospital, Capital Medical University, Beijing 100730, China

## Abstract

**Introduction:**

Antimicrobial peptides and proteins (AMPs) constitute the first line of defense against pathogenic microorganisms in the airway. The association between AMPs and chronic rhinosinusitis with nasal polyps (CRSwNP) requires further investigations. This study is aimed at investigating the expression and regulation of major dysregulated AMPs in the nasal mucosa of CRSwNP.

**Methods:**

The expression of AMPs was analyzed in nasal tissue from patients with eosinophilic (E) CRSwNP and nonECRSwNP and healthy subjects using RNA sequencing. The 10 most abundant AMPs expressed differentially in CRSwNP patients were verified by real-time PCR, and of these, the expression and regulation of secretory leukoprotease inhibitor (SLPI) and clusterin (CLU) were investigated further.

**Results:**

The 10 most abundant AMPs expressed differentially in CRSwNP compared to healthy control, regardless of subtypes, included BPIFA1, BPIFB1, BPIFB2, CLU, LTF, LYZ, and SLPI, which were downregulated, and S100A8, S100A9, and HIST1H2BC, which were upregulated. ELISA and immunofluorescence confirmed the decreased expression of SLPI and CLU levels in CRSwNP. SLPI is expressed in both nasal epithelial cells and glandular cells, whereas CLU is mainly expressed in glandular cells. AB/PAS staining further demonstrated that both SLPI and CLU were mainly produced by mucous cells in submucosal glands. Furthermore, the numbers of submucosal glands were significantly decreased in nasal polyp tissue of CRSwNP compared to nasal tissue of controls. SLPI was downregulated by TGF-*β*1 and IL-4 in cultured nasal tissues *in vitro*, while CLU expression was inhibited by TGF-*β*1. Glucocorticoid treatment for 2 weeks significantly increased the expression of all downregulated AMPs, but not LYZ. Additionally, budesonide significantly increased the expression of SLPI and CLU in cultured nasal tissues.

**Conclusion:**

The expression of major antimicrobial proteins is significantly decreased in nasal tissue of CRSwNP. The expression of SLPI and CLU is correlated with the numbers of submucosal glands and regulated by inflammatory cytokines and glucocorticoids.

## 1. Introduction

Chronic rhinosinusitis (CRS) is a common inflammatory disease of nasal and sinus mucosa, which affects 8% of the Chinese population and 10.9% of the European population, and exerts a heavy socioeconomic burden [1]. CRS is classified into 2 phenotypes based on the presence or absence of nasal polyps: chronic rhinosinusitis with nasal polyps (CRSwNP) and chronic rhinosinusitis without nasal polyps (CRSsNP) [2]. Patients with CRSwNP tend to develop more severe symptoms and have higher rates of asthma comorbidity and postoperative recurrence, compared to CRSsNP [3].

Considering the heterogeneous nature of the disease, CRSwNP can further be classified into 2 subgroups based on the eosinophil infiltration status: eosinophilic CRSwNP (ECRSwNP) and noneosinophilic CRSwNP (nonECRSwNP), which have distinct immune-inflammatory characteristics [4]. ECRSwNP presents a Th2 dominant inflammatory pattern, while nonECRSwNP is characterized by Th1/17 inflammation with a better prognosis [5].

The pathogenesis of CRSwNP is associated with complex interactions between inflammatory triggers and host inflammatory pathways [2]. Airway epithelium and submucosal glands play a pivotal role in secreting a variety of host defense molecules, especially a vast arsenal of antimicrobial peptides and proteins (AMPs) such as lysozyme (LYZ), defensins (DEF), lactoferrin (LTF), and S-100 proteins, which are essential for defense against a broad spectrum of pathogenic microorganisms [6, 7]. Several studies have indicated that the expression of AMPs is associated with the innate immune system [8–10]. Moreover, studies have also demonstrated the association between AMPs and airway diseases such as CRS, chronic obstructive pulmonary disease (COPD), bronchiectasis, and allergic asthma [11–14]. Indeed, one recent study indicated that the expression of AMPs was associated with airway inflammation, bacterial colonization, and exacerbation in COPD patients [15]. Although there is evidence that some AMPs are upregulated in CRS patients [16, 17], more recent evidence has indicated that some antimicrobial factors such as palate, lung, and nasal epithelium clone (PLUNC, also named BPIFA1) and sterol O-acyltransferase 1 may be downregulated in patients with CRSwNP [18, 19]. However, the differential expression of certain AMPs in CRSwNP is still controversial [20, 21].

The contribution of AMPs in defending against pathogens depends on their concentration and activity [22]. However, to date, there is still no clear understanding on the most abundant AMPs that associated with CRSwNP in nasal tissues. Thus, the present study is aimed at investigating the major AMPs which were dysregulated in the nasal mucosa of patients with CRSwNP compared to healthy control subjects. In the present study, employing RNA-sequencing technology, we identified the most abundant AMPs that were dysregulated in CRSwNP compared to healthy control subjects in the nasal mucosa. Among the 10 most abundant AMPs, secretory leukoprotease inhibitor (SLPI), a major antielastase barrier of the respiratory tract with antibacterial, antiviral, and anti-inflammatory properties [23], and clusterin (CLU), a secretory glycoprotein that acts as a cellular biosensor for oxidative stress and binds to some bacterial and bacterial proteins [24, 25], were further investigated for expression and regulation.

## 2. Materials and Methods

### 2.1. Patients and Study Design

A total of 96 adult subjects, including 40 patients with ECRSwNP, 22 patients with nonECRSwNP, and 34 healthy control subjects, were enrolled into the study. The diagnosis of CRSwNP was strictly consistent with the European Position Paper on Rhinosinusitis and Nasal Polyps 2012 [2]. The diagnosis of asthma and AR were consistent with prior medical history and serum sIgE results. The definitions of both ECRSwNP and nonECRSwNP were in accordance with a previous study [26]. Patients with acute upper or lower respiratory tract infections, patients who were treated with antibiotics or glucocorticoids in four weeks prior to the enrolment of the study, patients with severe uncontrolled systemic diseases, including active systemic/immunologic disease (such as systemic lupus erythematosus, inflammatory bowel disease, and nephritis), and pregnant women were excluded. Control subjects were recruited from those who underwent septoplasty surgery merely because of anatomic variation but without any rhinosinusitis. No medication was used prior to sampling.

A total of 18 CRSwNP patients underwent a 2-week oral glucocorticoid treatment (24 mg methylprednisolone, QD) prior to surgery. Polyp biopsies originated from the middle meatus of patients with CRSwNP were performed both before and after the oral glucocorticoid treatment. Postglucocorticoid polyp biopsies and uncinate mucosal tissue from healthy control subjects were collected during surgery. Detailed demographic and clinical information on smoking history, comorbidity of allergic rhinitis, and asthma and atopic data were collected on each participant on enrolment. Detailed information on demographical and clinical characteristics of subjects is provided in Table S1 and S2.

The study was approved by the Medical Ethics Committee of Beijing Tongren Hospital and Beijing Institute of Otolaryngology, and all participants provided written informed consent before entry into the study.

### 2.2. RNA Isolation and RNA Sequencing

Nasal tissue samples of eosinophilic CRSwNP (*n* = 6), nonECRSwNP (*n* = 6), and healthy control (*n* = 6) were performed RNA sequencing. Briefly, total RNA was extracted and purified with an RNeasy Kit (Qiagen, Hilden, Germany) according to the manufacturer's instructions. The quantity and quality of the isolated RNA were determined with a NanoDrop 2000 Spectrophotometer (Thermo Fisher Scientific) and 2100 TapeStation Automated Electrophoresis System (Agilent Technologies). RNA sequencing was performed on the Illumina HiSeq platform, and 150 bp paired-end reads were generated by Novogene Bioinformatics Technology Cooperation (Beijing, China).

### 2.3. RNA Sequencing Data Analysis and the Expression of AMPs

The analyses of RNA sequencing data were performed as previously described [26]. An adjusted *P* < 0.05 plus fold change > 2 was used as the cut-off for significantly differentially expressed mRNAs. All the genes included in Gene Ontology term “antimicrobial humoral response” (accession number 0019730) were determined for significantly differential expression between the eosinophilic CRSwNP group and the control group and between the noneosinophilic CRSwNP group and the control group. Transcripts Per Million (TPM) of mRNAs were used to represent the RNA abundance. The 10 most abundant AMPs differentially expressed in CRSwNP compared to healthy controls were selected for further analyses.

### 2.4. Histologic Staining

In total, samples from 18 subjects (6 ECRSwNP patients, 6 nonECRSwNP patients, and 6 control subjects) were processed for preparation of serial sections of Alcian blue/periodic acid-Schiff (AB/PAS) staining, immunohistochemistry, and immunofluorescence staining, and samples from 33 subjects (13 ECRSwNP, 7 nonECRSwNP, and 13 control subjects) were processed for hematoxylin and eosin (H&E) and AB/PAS staining for assessment of glands and goblet cell hyperplasia in the samples. Immunohistochemistry and immunofluorescent staining for BPIFB2 (13461-2-AP, Proteintech, China), CLU (ab92548, Abcam, UK), and SLPI (ab17157, Abcam, UK) were performed using commercially available kits according to the manufacturer's instructions.

### 2.5. RNA Isolation, Reverse Transcription, and Quantitative Real-Time PCR

Total RNA was extracted using the MiniBEST Universal RNA Extraction Kit (TaKaRa Biotechnology, Dalian, China), in accordance with the manufacturer's instructions. The quality and quantity of the total RNA were assessed with the NanoDrop 2000 system (Thermo Fisher Scientific, Waltham, Mass), and cDNA was subsequently synthesized using the PrimeScript RT Master Mix (TaKaRa Biotechnology, Dalian, China). Quantitative real-time PCR (qRT-PCR) was then performed with the SYBR Green method (TaKaRa Biotechnology) to evaluate the amount of mRNA for the AMPs of interest. The primer sequences for each gene used in the study are listed in Table S3.

### 2.6. Nasal Secretion and ELISA

Nasal secretion from 21 patients with ECRSwNP, 11 patients with nonECRSwNP, and 16 healthy volunteers was obtained and processed as previously described [27]. Briefly, the nasal secretion was collected by inserting a sponge pack in the common meatus for 5 minutes. Then, the sponge was collected in a centrifugal tube and added 1 milliliter of normal saline. After incubation at 4°C for 2 hours, tubes were centrifugated with the speed of 1500 rpm for 8 minutes at 4°C. Supernatants were collected and stored at -80°C for further analysis. Protein levels of SLPI and CLU in nasal secretion were measured using SimpleStep ELISA kits ab263890 and ab174447, from Abcam (Cambridge, UK) according to the manufacturer's instructions.

### 2.7. Nasal Tissue and Cell Culture and Stimulation

The nasal tissue culture and stimulation were performed as previous study [28]. Nasal tissues collected from patients with CRSwNP which consist of nasal mucosa and submucosa were cut thoroughly in RPMI 1640 medium. Tissue fragment was achieved by passing through a mesh and then was resuspended in the culture medium, ready for further stimulating experiments. Also, A549 lung adenocarcinoma tumor cells were cultured as submersion cultures in DMEM until passaged. Nasal tissue fragment suspension and the passaged A549 cultures were stimulated by incubation for 48 hours in the presence of budesonide (1 *μ*g/mL) and several cytokines, including IL-4 (50 ng/mL, PeproTech), IL-13 (50 ng/mL, R&D systems), IL-1*β* (10 ng/mL, R&D Systems), IFN-*γ* (50 ng/mL, R&D Systems), and TGF-*β*1 (10 ng/mL, R&D Systems), and at the end of this incubation period, the cells from each culture were harvested for analysis of SLPI and CLU by real-time PCR as detailed above.

### 2.8. Statistical Analysis

Data were analyzed using the SPSS V.19 software package (IBM Corp., Armonk, New York, USA). Graphs were performed with GraphPad Prism V.7.0 software (GraphPad Software, San Diego, CA, USA). Descriptive statistics were used for general information of the study population, and distribution of the data was assessed for normality. Results were expressed as mean ± standard deviation (SD). The Mann-Whitney *U* test or Student *t* test was used to analyze the differences depending on the normality of data distribution. For paired data, the Wilcoxon rank test was performed. A value of *P* < 0.05 was considered to be significant.

## 3. Results

### 3.1. Differential Expression of AMPs between CRSwNP and Healthy Controls

**Figure 1:**
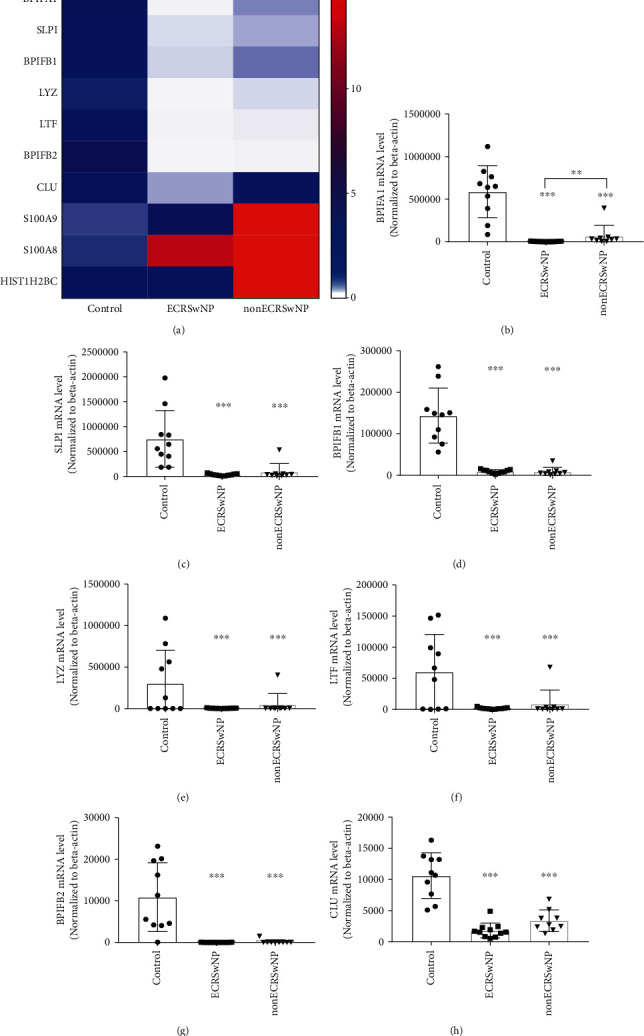
Expression of top abundant AMPs in nasal tissues. (a) Expression of the 10 most abundant AMPs in nasal tissues from ECRSwNP, nonECRSwNP, and healthy controls identified by RNA sequencing. The color coding of heat maps represents the gene expression level normalized to the control group, which is calculated based on Transcripts Per Million (TPM). A significant difference in the expression of BPIFA1 was found between the ECRSwNP and nonECRSwNP groups. (b–h) The expression of AMPs (BPIFA1, SLPI, BPIFB1, LYZ, LTF, BPIFB2, and CLU) in nasal tissue of ECRSwNP, nonECRSwNP, and healthy controls verified by real-time PCR. ^∗^*P* < 0.05, ^∗∗^*P* < 0.01, and ^∗∗∗^*P* < 0.001. AMPs: antimicrobial peptides and proteins; CRSwNP: chronic rhinosinusitis with nasal polyps; ECRSwNP: eosinophilic CRSwNP; BPIFA: BPI fold-containing family A member; BPIFB: BPI fold-containing family B member; CLU: clusterin; LTF: lactoferrin; LYZ: lysozyme; SLPI: secretory leukoprotease inhibitor.

The 10 most abundant AMPs expressed differentially in nasal tissues of ECRSwNP and nonECRSwNP patients and control subjects, based on RNA abundance determined by average TPM, are shown in Figure 1(a) and Table S4 (Supplementary Materials). The mRNA expression of BPIFA1, BPIFB1, BPIFB2, LYZ, LTF, SLPI, and CLU was significantly downregulated in both the ECRSwNP (*P* < 0.05) and nonECRSwNP (*P* < 0.05) groups compared to the control group, whereas the expression of S100A8, S100A9, and HIST1H2BC was significantly upregulated in both the ECRSwNP (*P* < 0.05) and nonECRSwNP (*P* < 0.05) groups compared with the control group. Indeed, the expression of BPIFA1 in the ECRSwNP group was also significantly lower than that in the nonECRSwNP group (*P* < 0.05; Figure 1(a)).

The downregulated expression of the various AMPs (BPIFA1, BPIFB1, BPIFB2, LYZ, LTF, SLPI, and CLU) was further verified by real-time PCR. As shown in Figures 1(b)–1(h), the expression levels of these AMPs were in accordance with the RNA sequencing data, showing significant decreases in both ECRSwNP and nonECRSwNP (*P* < 0.001, respectively).

In order to evaluate the possible bias on the expression of AMPs due to anatomical differences, we collected nasal mucosa samples from different anatomical sites including uncinate process, inferior turbinate, and middle turbinate from control subjects. As shown in Figure S1, there is no difference in the expression of SLPI and CLU between different anatomical sites (uncinate process, inferior turbinate, and middle turbinate), respectively.

### 3.2. Expression of SLPI and CLU at Protein Level in Nasal Tissues and Secretions

**Figure 2:**
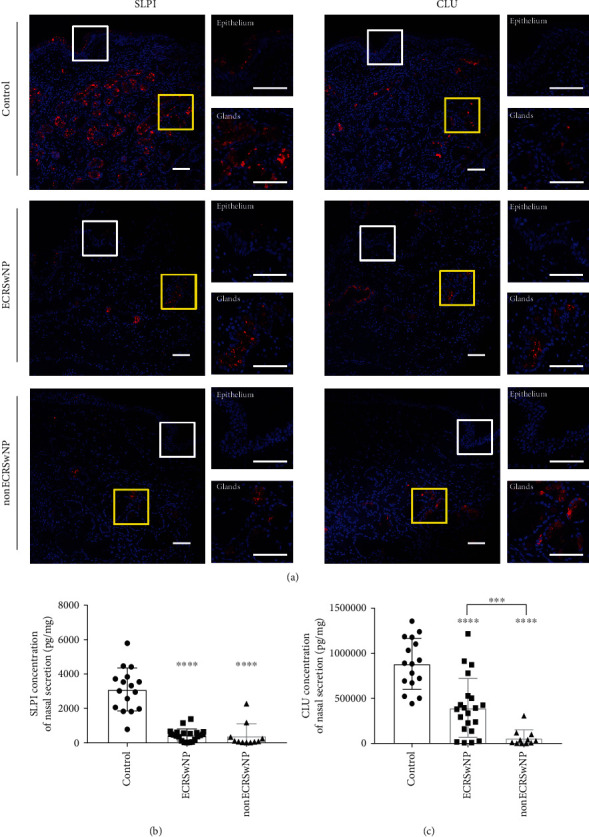
Expression of SLPI and CLU at the protein level in nasal tissues of CRSwNP. (a) Immunofluorescence staining of SLPI and CLU in nasal tissues from ECRSwNP, nonECRSwNP, and healthy controls. Epithelium area and submucosal glands were displayed at high magnification. Bars = 25 *μ*m. (b, c) The expression of SLPI (b) and CLU (c) in nasal secretions of ECRSwNP, nonECRSwNP, and healthy controls detected by ELISA. ^∗^*P* < 0.05, ^∗∗^*P* < 0.01, and ^∗∗∗^*P* < 0.001. CRSwNP: chronic rhinosinusitis with nasal polyps; ECRSwNP: eosinophilic CRSwNP; nonECRSwNP: noneosinophilic CRSwNP; CLU: clusterin; SLPI: secretory leukoprotease inhibitor.

Among the seven downregulated AMPs in CRSwNP, the association between SLPI and CRSwNP and the association between CLU and CRSwNP are still unclear. Immunofluorescence results demonstrated that SLPI was expressed in both nasal epithelial cells and glandular cells, while CLU was mainly expressed in glandular cells (Figure 2(a)). Immunofluorescence also showed decreased trends of SLPI expression and CLU expression in samples from ECRSwNP and nonECRSwNP patients, compared to samples from healthy controls. Moreover, ELISA assays to determine the expression of SLPI and CLU in nasal secretions further confirmed the decreased expression of SLPI and CLU in both the ECRSwNP and nonECRSwNP groups compared to healthy controls (*P* < 0.001, respectively; Figures 2(b) and 2(c)). Additionally, CLU expression levels in nasal secretions from nonECRSwNP patients were much lower than in nasal secretions from ECRSwNP patients (Figure 2(c)).

**Figure 3:**
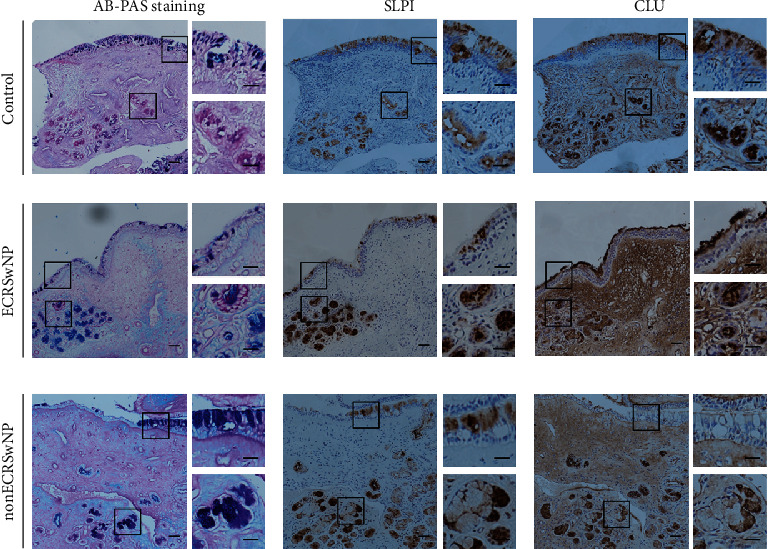
Evaluation of sequential staining for AB/PAS, SLPI, and CLU in nasal tissues. AB/PAS staining and immunohistochemical staining for SLPI and CLU were performed on serial sections of nasal tissues from ECRSwNP, nonECRSwNP, and healthy controls. Bars = 50 *μ*m. ECRSwNP: eosinophilic CRSwNP; nonECRSwNP: noneosinophilic CRSwNP; CLU: clusterin; SLPI: secretory leukoprotease inhibitor.

AB/PAS staining and immunohistochemical staining were performed on serial sections of nasal tissues from ECRSwNP, nonECRSwNP, and healthy control subjects to further investigate the cellular localization of SLPI and CLU in submucosal glands. BPIFB2 was used as a positive control, as previous evidence has indicated that BPIFB2 was restricted to regions that stained with AB/PAS [29]. The present study demonstrated that the expression of both SLPI and CLU was restricted to regions that stained with AB/PAS, indicating that both SLPI and CLU were mainly produced by mucous cells of the submucosal glands (Figure 3). Furthermore, BPIFB2 staining was also restricted to AB/PAS staining (Figure S2), as has been demonstrated previously [29].

### 3.3. Glucocorticoid Treatment Restored AMP Expression

**Figure 4:**
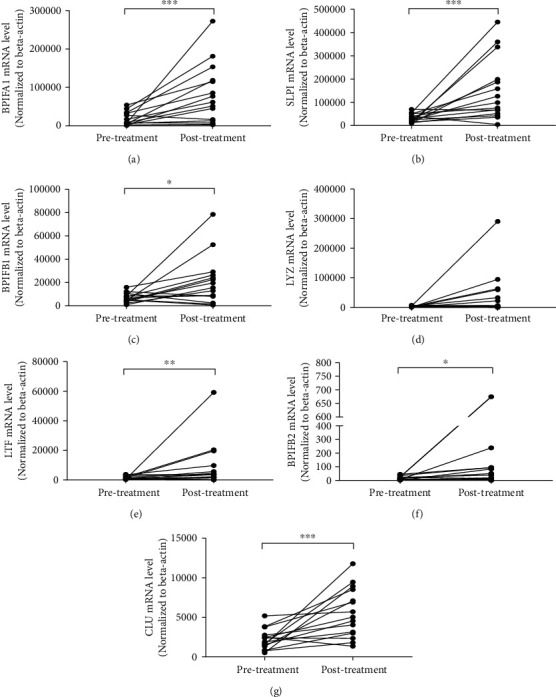
The expression of AMPs in nasal tissues of CRSwNP in response to glucocorticoid treatment. Patients with CRSwNP were treated with 2-week oral glucocorticoid. The expression of AMPs (BPIFA1, SLPI, BPIFB1, LYZ, LTF, BPIFB2, and CLU) was detected in nasal polyp tissues before and after glucocorticoid treatment by real-time PCR. ^∗^*P* < 0.05, ^∗∗^*P* < 0.01, and ^∗∗∗^*P* < 0.001. AMPs: antimicrobial peptides and proteins; CRSwNP: chronic rhinosinusitis with nasal polyps; BPIFA: BPI fold-containing family A member; BPIFB: BPI fold-containing family B member; CLU: clusterin; LTF: lactoferrin; LYZ: lysozyme; SLPI: secretory leukoprotease inhibitor.

Real-time PCR analysis demonstrated that glucocorticoid treatment for 2 weeks significantly increased the expression of BPIFA1 (*P* < 0.001), BPIFB1 (*P* < 0.05), BPIFB2 (*P* < 0.05), LTF (*P* < 0.01), SLPI (*P* < 0.001), and CLU (*P* < 0.001), but not LYZ, in nasal tissue of patients with CRSwNP (Figure 4). The expression of SLPI was increased around 5-fold and the expression of CLU about 2.5-fold from baseline levels, following glucocorticoid treatment.

### 3.4. Reduced Submucosal Gland Numbers in CRSwNP

**Figure 5:**
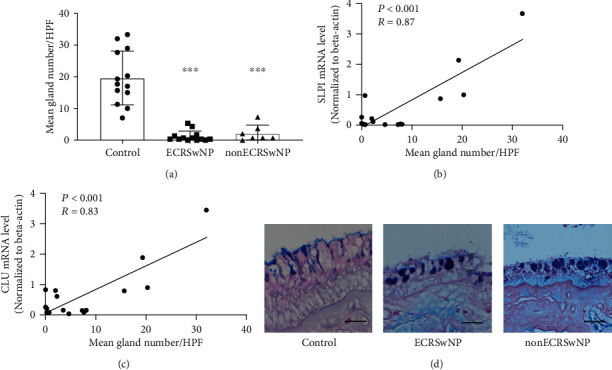
The expression of SLPI and CLU is correlated with the numbers of submucosal glands. (a) The number of submucosal glands per high power field (×200 magnifications) in the nasal mucosa of ECRSwNP, nonECRSwNP, and healthy controls. (b, c) Correlations between the gland counts and the expression of SLPI and CLU. (d) AB/PAS staining shows goblet cell hyperplasia in the epithelial mucosa of ECRSwNP, nonECRSwNP, and healthy controls. Bars = 50 *μ*m. ^∗∗∗^*P* < 0.001. ECRSwNP: eosinophilic CRSwNP; nonECRSwNP: noneosinophilic CRSwNP.

To explore the reasons for the decreased expression of AMPs, the numbers of submucosal glands were evaluated in CRSwNP and control samples. As shown in Figure 5(a), based on the AB/PAS and HE staining, the numbers of submucosal glands per high power field were significantly reduced in polyp tissues from ECRSwNP (1.18 ± 1.71; *P* < 0.001) and nonECRSwNP (2.14 ± 2.59; *P* < 0.001) compared to controls (19.64 ± 8.48); however, there was no significant difference between the ECRSwNP and nonECRSwNP (*P* = 0.24) groups. Furthermore, significant correlations were found between the counts of submucosal glands and the expression of SLPI (*R* = 0.87, *P* < 0.001) and CLU (*R* = 0.83, *P* < 0.001; Figures 5(b) and 5(c)). Goblet cell hyperplasia was prevalent in the nasal epithelium in nasal polyps regardless of eosinophilic infiltration (Figure 5(d)).

### 3.5. CLU and SLPI Were Regulated by Inflammatory Cytokines

**Figure 6:**
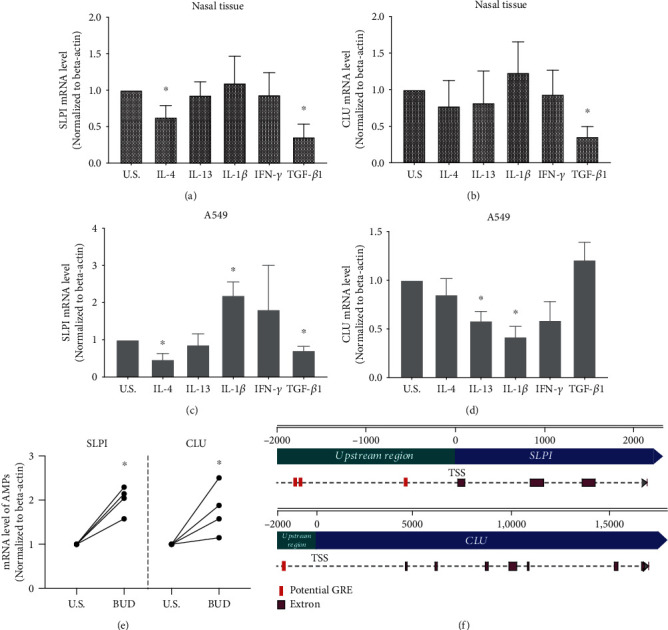
Regulation of SLPI and CLU by cytokines and glucocorticoid. (a–d) The expression level of SLPI (a, c) and CLU (b, d), detected by real-time PCR, in cultured human nasal tissues and A549 cells after 48 hours of stimulation by cytokines or unstimulated control. (e) The upregulated mRNA level of SLPI and CLU in cultured nasal tissues after 48 hours of stimulation by budesonide. (f) Potential glucocorticoid response element in the upstream region of SLPI and CLU. ^∗^*P* < 0.05; CLU: clusterin; SLPI: secretory leukoprotease inhibitor; BUD: budesonide; U.S.: unstimulated control; GRE: glucocorticoid response element; TSS: transcription start.

The effects of IFN-*γ* (Th1 cytokine), IL-4 and IL-13 (Th2 cytokines), IL-1*β* (proinflammatory cytokine), and TGF-*β*1 (regulatory T-cell cytokine) were investigated on the expression of SLPI and CLU in cultured nasal tissues and A549 cells. Stimulation for 48 hours by different cytokines demonstrated that the expression of SLPI was significantly downregulated by the stimulation of IL-4 and TGF-*β*1 (*P* < 0.05, Figure 6(a)), while CLU was downregulated by TGF-*β*1 (*P* < 0.05, Figure 6(b)) in cultured nasal tissues. In addition, SLPI was significantly downregulated by TGF-*β*1 and IL-4 (*P* < 0.05; Figure 6(c)), while CLU expression was inhibited by IL-13 and IL-1*β* in cultured A549 cells (*P* < 0.05; Figure 6(d)).

### 3.6. Glucocorticoid Upregulated the Expression of SLPI and CLU

To further investigate the regulatory effect of glucocorticoids on AMP expression, nasal tissues were cultured and stimulated with budesonide (1 *μ*g/mL). Then, the expression of SLPI and CLU was evaluated by real-time PCR. Both SLPI and CLU were significantly upregulated by budesonide in cultured nasal tissues (*P* < 0.05, Figure 6(e)). As the effects of glucocorticoid are usually mediated by glucocorticoid response elements (GREs) that serve as a DNA binding site for glucocorticoid receptor, GREs were predicted in 2 kb upstream of the transcription initiation site of the SLPI gene and CLU gene, respectively. The results showed 3 canonical GRE half-sites (AGAACA) located upstream of the transcription initiation site of the SLPI gene while CLU had 1 GRE half-site (Figure 6(f)).

## 4. Discussion

The present study has investigated the 10 most abundant AMPs that are differentially expressed in nasal tissues of CRSwNP compared to healthy control and demonstrated that most of these were downregulated in CRSwNP, which as a consequence might potentiate chronic nasal inflammation and contribute to the pathogenesis of CRSwNP. Furthermore, to our knowledge, our study is the first to have identified the decreased expression of SLPI and CLU in CRSwNP and illustrated the expression pattern of these AMPs in nasal tissue. Moreover, our study has demonstrated that the expression of CLU and SLPI might be regulated by cytokines and glucocorticoid.

CRSwNP is a predominantly chronic inflammatory disease associated with dysregulated host-microbial interactions [29]. AMPs play fundamental roles in innate host defense by inhibiting proliferation of the microbiome and trapping and killing of pathogens [30]. Our finding that the major AMPs were decreased in nasal tissues of patients with CRSwNP is in accordance with previous studies investigating the expression of a variety of AMPs. For example, Wei and colleagues compared the expression of short palate, lung, and nasal epithelium clone 1 (SPLUNC1; also known as BPIFA1) in polyp tissue of ECRSwNP/nonECRSwNP patients with an uncinate process in controls and reported that the expression level of BPIFA1 was significantly lower in nasal polyps than in control uncinate tissues [18]. Similarly, other studies have also reported profound decreases in the expression of BPIFA1 and BPIFB2 in nasal polyps [31] and LTF in nasal tissue of CRSwNP patients [32]. However, studies investigating the expression of LYZ in CRS patients have provided conflicting data. While some researchers have shown LYZ expression to be elevated in CRS [20, 33], others have reported decreased expression of LYZ [21].

CRSwNP is a multifactorial and highly heterogeneous disease, which can further be classified into ECRSwNP and nonECRSwNP. ECRSwNP was the predominant CRSwNP subtype in Western countries, whereas nonECRSwNP had a higher prevalence in East Asia such as China [34]. Numerous studies indicated that the two CRSwNP subtypes exhibited distinct clinical characteristics, pathological characteristics, and inflammatory characteristics [35–37]. Our findings indicated that BPIFA1 was differentially impaired between ECRSwNP and nonECRSwNP, which was in consistent with the previous study [18]. Although there is no differential expression of CLU between nasal tissues from ECRSwNP and nonECRSwNP at mRNA level, we observed that CLU expression in nasal secretions from nonECRSwNP was much lower than from ECRSwNP at protein levels. Therefore, a lower CLU expression might be associated with the development of nonECRSwNP. However, our findings indicated an overall significantly decreased expression of AMPs in CRSwNP, regardless of the eosinophilic subtypes, which implies a general mechanism for potentiation of the pathogenesis of CRSwNP.

To our knowledge, our study is also the first to confirm the association between specifically SLPI and CLU and CRSwNP. In particular, our study has shown that SLPI, a neutrophil elastase inhibitor with broad-spectrum antimicrobial function [38], which can be secreted by airway epithelium cells, macrophages, and neutrophils, was mainly produced by nasal epithelial cells and glandular cells in nasal tissue. Within the submucosal glands, SLPI was mainly produced by the mucous cells. Furthermore, our study demonstrated that SLPI was significantly decreased at both mRNA and protein levels in the nasal tissues of CRSwNP patients, regardless of the eosinophilic subtypes. However, one previous study has shown that the expression of SLPI was increased in the nasal mucosa from a subgroup of bacterial biofilm-positive CRS patients compared to healthy controls, whereas no difference was found between the 1subgroup of bacterial biofilm-negative CRS patients and the control group [33]. In view of the finding from the present study that the numbers of submucosal glands in nasal polyps were also significantly decreased in the CRSwNP patients, we speculate that the decreased expression of SLPI in CRS patients is likely to be primarily associated with nasal polyposis in CRS.

CLU is a secretory glycoprotein, which is known to act as a cellular biosensor for oxidative stress, as well as an antimicrobial protein due to its ability to bind to some bacteria and bacterial proteins [25, 39, 40]. Sol and colleagues have reported that the levels of sputum CLU were significantly lower in asthmatic patients than healthy controls, and moreover, the levels of CLU were associated with asthma severity [41]. Another study has indicated that CLU was associated with mucus secretion in human airway epithelial cells via activation of NF-*κ*B [42]. The present study has indicated a relationship between CLU and CRSwNP, with significantly decreased CLU level in CRSwNP.

The finding from the current study that the numbers of submucosal glands were significantly lower in nasal polyp tissue than in control uncinate tissue regardless of the eosinophilic infiltration was consistent with the previous study that the submucosal glandular density decreased in nasal polyps than the surrounding ethmoidal tissue [43]. As CLU and SLPI were both found to be localized in the mucous cells of submucosal glands, it is possible that the decreased number of submucosal glands may be an important reason for the reduced expression of AMPs seen in the present study, as well as that indicated by the study on BPIFA1 in CRSwNP [44]. Two studies [4, 18] reported a higher number of submucosal glands in nonECRSwNP versus ECRSwNP. Although our results showed an increasing trend of gland number in nonECRSwNP versus ECRSwNP, the difference was not statistically significant. One possible reason could be due to the geographical difference. The above two studies were conducted in southern China (Wuhan and Guangzhou city, respectively), while our cohort is from Beijing. It is widely accepted that the immunological characteristics of nasal polyps from Beijing tend to be more severe than those from southern China [45].

We further showed that SLPI and CLU can be downregulated by several inflammatory cytokines that play critical roles in the pathogenesis of CRSwNP. TGF-*β*1 has been shown to play a key role in the development of epithelial-to-mesenchymal transition [46, 47], in which differentiated nasal epithelial cells lose their normal morphology and function, then might thus affect the expression of SLPI and CLU. Our findings are in consistent with a previous report that TGF-*β*1 significantly downregulated the expression of SLPI in corneal and conjunctival epithelial cells [48, 49]. Elevated expression of Th2 cytokines such as IL-4 and IL-13 is a predominant characteristic in CRSwNP, especially in ECRSwNP. The Th2 environment might contribute to the decreased expression of SLPI, which is in consistent with previous studies that Th2 cytokines reduced the expression of certain AMPs in nasal epithelial cells [14, 18]. However, Th2 cytokines might not contribute to the decreased expression of CLU in nasal tissues of CRSwNP as indicated by our findings from cultured nasal tissue. Therefore, a different response towards inflammatory cytokines might be responsible for the differentiated expression of SLPI and CLU, respectively.

Glucocorticoids are the first-line medical treatment for CRSwNP, which significantly improve nasal symptoms and reduced the size of NPs [50]. Our study has shown that AMP levels were significantly increased after 2-weeks' treatment with oral glucocorticoid, suggesting that glucocorticoids might be also capable of enhancing the innate immune response by upregulating the expression of AMPs. These findings are in accordance with a previous study on BPIFA1, which has been shown to be regulated by glucocorticoid treatment [18]. Thus, AMPs produced by nasal mucosa might not only contribute to the pathogenesis of CRSwNP but also respond to clinical treatment. A Spanish research group reported that 2-week corticosteroid treatment could significantly increase the numbers of submucosal glands [51], which could be one important reason for the increased expression of AMPs. We also confirmed the regulatory effect of glucocorticoids on SLPI and CLU *in vitro*. In addition, we found GRE that serves as a DNA-binding site for glucocorticoid receptor located upstream of the transcription initiation site of both the SLPI gene and the CLU gene, respectively. These GRE half-sites positioned nearby the promoters of SLPI and CLU suggest a role for sequence-specific binding of glucocorticoid receptor which might drive the transcription [52, 53].

The findings of the current study, however, are somewhat limited. Firstly, the abundance of AMPs in nasal mucosa was represented by RNA abundance in this study. The expression abundance of AMPs needs to be investigated at the protein level in order to confirm these findings. Secondly, the study is limited in that the function of SLPI and CLU was not investigated to clarify the specific roles of these dysregulated proteins in the pathogenesis of CRSwNP. Thirdly, the relationship between AMP levels and microbial colonization and infection in nasal mucosa was not investigated, which might have provided a generally better understanding of the impact of these proteins on the microbiome in CRS. Finally, although some studies have shown the expression of AMPs S100A8, S100A9, and HIST1H2BC to upregulate in CRSwNP [54, 55], this was not verified in the present study.

## 5. Conclusion

Taken together, our data indicate that major AMPs including SLPI and CLU are decreased in nasal tissues of CRSwNP patients. Decreased numbers of submucosal glands and regulation by inflammatory cytokines might explain the reduced expression of SLPI and CLU in CRSwNP. Glucocorticoid treatment contributes to increasing SLPI and CLU expression. These findings help to better understand the association between dysregulation of major AMPs and pathogenesis of CRSwNP, as well as provide potential targets for the development of novel therapies for CRSwNP.

## Data Availability

The data used in this study are shown as supplementary files.

## Supplementary Materials

Supplementary Materials Table S1: demographical characteristics of subjects. Table S2: demographical characteristics of subjects who underwent glucocorticoid treatment. Table S3: primers used for real-time PCR analysis. Table S4: expression of top 10 abundant AMPs in nasal tissues from CRSwNP and healthy control identified by RNA sequencing. Figure S1: expression of CLU (A) and SLPI (B) in nasal tissues from uncinate process, inferior turbinate, and middle turbinate. CLU: clusterin; SLPI: secretory leukoprotease inhibitor. Figure S2: evaluation of sequential staining for AB/PAS and BPIFB2. AB/PAS staining and immunohistochemical staining for BPIFB2 were performed on serial sections of nasal tissues from ECRSwNP, nonECRSwNP, and controls. Bars = 50 *μ*m. CRSwNP: chronic rhinosinusitis with nasal polyps; ECRSwNP: eosinophilic CRSwNP; BPIFB2: BPI fold-containing family B member 2.
