# Supplementary material for: Reduced Expression of Antimicrobial Protein Secretory Leukoprotease Inhibitor and Clusterin in Chronic Rhinosinusitis with Nasal Polyps
Source: J Immunol Res. 2021 Jan 7;2021:1057186. doi: 10.1155/2021/1057186 (PMC7810533; doi:10.1155/2021/1057186)

**Supplementary Materials**

**Supplemental Tables**

**Table S1. Demographical characteristics of subjects**

| Methodology used | Control | ECRSwNP | nonECRSwNP | *P* value |
| --- | --- | --- | --- | --- |
| RNA-sequencing for nasal tissues | | | | |
| Subjects (%) | 6(33.3%) | 6(33.3%) | 6(33.3%) |  |
| Gender, male (%) | 3(16.7%) | 4(22.2%) | 3(16.7%) | 0.988 |
| Age (years±SD) | 49.50±16.17 | 44.67±7.53 | 41.83±15.87 | 0.631 |
| Atopy (%) | 4(22.2) | 2(11.1%) | 2(11.1%) | 0.477 |
| Asthma (%) | 0 | 4(22.2%) | 0 | 0.006 |
| Smoking history (%) | 0 | 1(5.6%) | 0 | 0.347 |
| Real time-PCR for nasal tissues | | | | |
| Subjects (%) | 10(31.3%) | 13(40.6%) | 9(28.1%) |  |
| Gender, male (%) | 7(21.9%) | 7(21.9%) | 7(21.9%) | 0.479 |
| Age (years±SD) | 48.80±12.94 | 41.23±9.23 | 46.22±14.33 | 0.319 |
| Atopy (%) | 6(18.8%) | 7(21.9%) | 3(9.4%) | 0.478 |
| Asthma (%) | 0 | 10(31.3%) | 1(3.1%) | 0.001 |
| Smoking history (%) | 1(3.1%) | 3(9.4%) | 1(3.1%) | 0.629 |
| IHC and IF |  |  |  |  |
| Subjects (%) | 6(33.3%) | 6(33.3%) | 6(33.3%) |  |
| Gender, male (%) | 4(22.2%) | 4(22.2%) | 4(22.2%) | 1.000 |
| Age (years±SD) | 49.50±16.17 | 44.67±7.53 | 41.83±15.87 | 0.631 |
| Atopy (%) | 2(11.1%) | 4(22.2%) | 4(22.2%) | 0.407 |
| Asthma (%) | 0 | 4(22.2%) | 0 | 0.006 |
| Smoking history (%) | 0 | 1(5.6%) | 0 | 0.347 |
| Glands counting by H&E and AB/PAS staining | | | | |
| Subjects (%) | 13(39.4%) | 13(39.4%) | 7(21.2%) |  |
| Gender, male (%) | 7(21.2%) | 7(21.2%) | 4(12.1%) | 0.988 |
| Age (years±SD) | 48.85±14.31 | 43±8.84 | 45.57±8.04 | 0.424 |
| Atopy (%) | 5(15.2%) | 8(24.2%) | 3(9.1%) | 0.473 |
| Asthma (%) | 0 | 10(30.3%) | 4(12.1%) | 0.003 |
| Smoking history (%) | 1(3.0%) | 2(6.1%) | 1(3.0%) | 0.819 |
| ELISA of nasal secretion | | | | |
| Subjects (%) | 16(33.3%) | 21(43.8%) | 11(22.9%) |  |
| Gender, male (%) | 7(14.6%) | 13(27.1%) | 9(18.8%) | 0.136 |
| Age (years±SD) | 28.56±4.16 | 42.05±14.88 | 51.18±11.33 | 0.001 |
| Atopy (%) | 1(2.1%) | 8(16.7%) | 3(6.3%) | 0.084 |
| Asthma (%) | 0 | 11(22.9%) | 4(8.3%) | 0.003 |
| Smoking history (%) | 0 | 6(12.5%) | 3(6.3%) | 0.062 |

**Table S2. Demographical characteristics of subjects underwent glucocorticoid treatment**

|  | CRSwNP |
| --- | --- |
| Subjects | 18 |
| Gender, male (%) | 10(55.6%) |
| Age (years±SD) | 41.33±11.58 |
| Atopy(%) | 7(38.9%) |
| Asthma (%) | 8(44.4%) |
| Smoking history (%) | 3(16.7) |

**Table S3. Primers used for real-time PCR analysis**

| Primer | Sequence |
| --- | --- |
| ACTB | F: 5’-CATGTACGTTGCTATCCAGGC -3’ |
|  | R: 5’-CTCCTTAATGTCACGCACGAT -3’ |
| BPIFA1 | F:5’-TGGAAAACCTTCCGCTCCTG -3’ |
|  | R:5’-ACTTTTCCAAGCAGTCCCCC -3’ |
| BPIFB1 | F:5’-CCAACTGATCGTGCTGGAAG -3’ |
|  | R:5’-AACCAGCCAATCCCAGAGTT -3’ |
| BPIFB2 | F:5’-CGTTTTTGAGAAGCCCCTGC -3’ |
|  | R:5’-GGGCGACATAGTGGAGGTTG -3’ |
| CLU | F:5’-CCATGTTCCAGCCCTTCCTT -3’ |
|  | R:5’-GTCATCGTCGCCTTCTCGTA -3’ |
| LTF | F:5’-CCCTGGTGCTGAAAGGAGAA -3’ |
|  | R:5’-GCACCAAACCACATTTGCCT -3’ |
| LYZ | F:5’-TTTCTGTTACGGTCCAGGGC -3’ |
|  | R:5’-ACACATCCAGTTTGCTAGGCT -3’ |
| SLPI | F:5’-TTGACACCCCAAACCCAACA -3’ |
|  | R:5’-CACATGCCCATGCAACACTT -3’ |

**Table S4. Expression of top 10 abundant AMPs in nasal tissues from CRSwNP and healthy control identified by RNA sequencing**

| **AMP genes** | **Control (TPM mean)** | **ECRSwNP (TPM mean)** | **nonECRSwNP (TPM mean)** | **Adjusted p-value ECRSwNP vs. Control** | **Adjusted p-value nonECRSwNP vs. Control** |
| --- | --- | --- | --- | --- | --- |
| BPIFA1 | 2128.16 | 20.14 | 311.71 | 5.04E-16 | 2.98E-08 |
| SLPI | 361.05 | 14.87 | 48.91 | 2.66E-22 | 1.91E-16 |
| BPIFB1 | 848.18 | 54.72 | 260.15 | 1.21E-11 | 5.10E-13 |
| LYZ | 220.17 | 2.32 | 15.52 | 1.18E-20 | 6.45E-11 |
| LTF | 653.35 | 8.64 | 12.32 | 3.83E-11 | 2.26E-14 |
| BPIFB2 | 118.14 | 0.31 | 0.52 | 5.21E-15 | 3.04E-15 |
| CLU | 50.63 | 6.70 | 38.34 | 6.45E-07 | 2.27E-03 |
| S100A8 | 0.10 | 0.42 | 9.92 | 1.54E-16 | 6.04E-20 |
| S100A9 | 0.02 | 0.19 | 4.91 | 2.55E-14 | 1.17E-17 |
| HIST1H2BC | 0.57 | 0.88 | 10.06 | 2.71E-03 | 5.92E-04 |

**Supplemental Figures**

**Figure S1.** Expression of CLU (A) and SLPI (B) in nasal tissues from uncinate process, inferior turbinate and middle turbinate. CLU, clusterin; SLPI, secretory leukoprotease inhibitor.


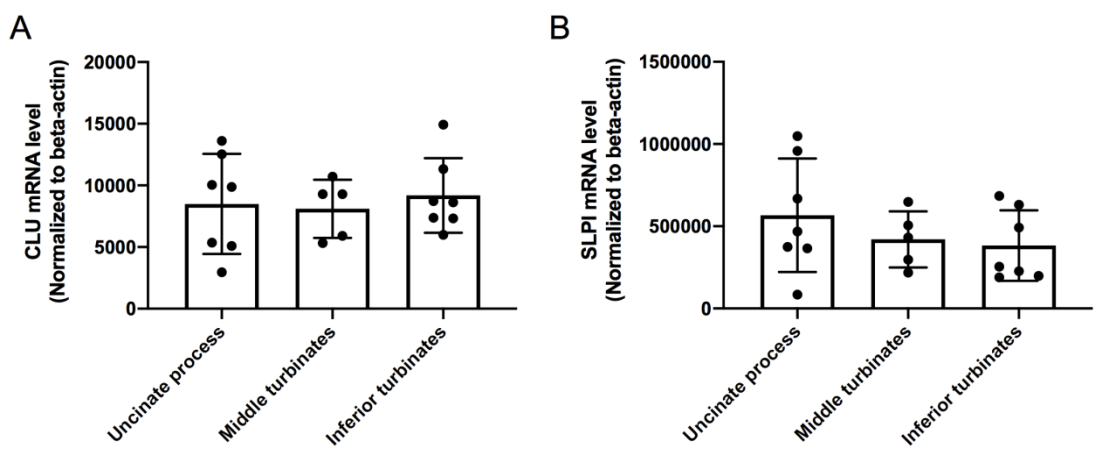


**Figure S2.** Evaluation of sequential staining for AB/PAS and BPIFB2. AB/PAS staining and immunohistochemical staining for BPIFB2 were performed on serial sections of nasal tissues from ECRSwNP, nonECRSwNP and controls. Bars=50μm. CRSwNP, chronic rhinosinusitis with nasal polyps; ECRSwNP, eosinophilic CRSwNP; BPIFB2, BPI fold-containing family B member 2.


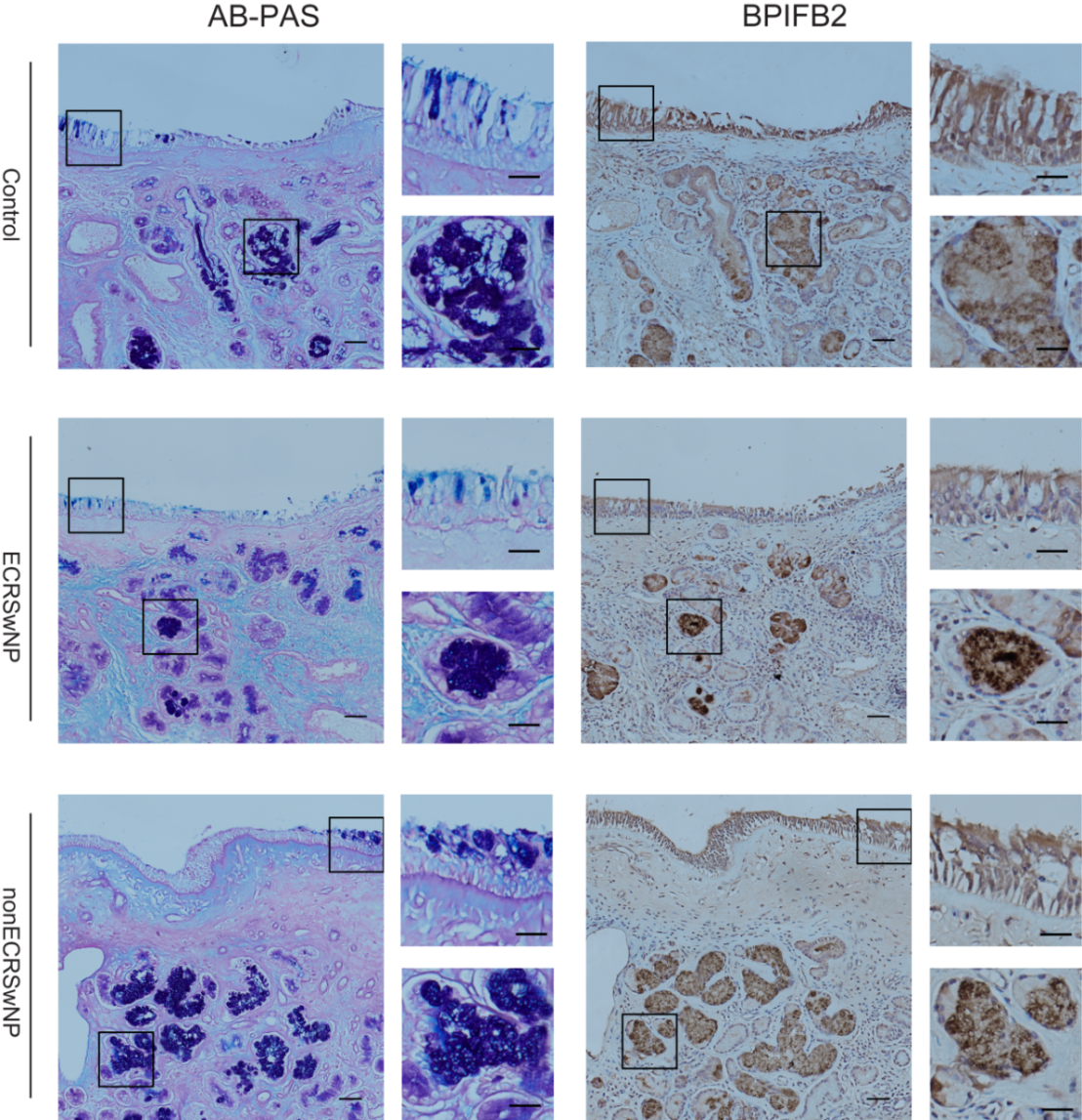

Supplement: Supplementary Materials — Table S1: demographical characteristics of subjects. Table S2: demographical characteristics of subjects who underwent glucocorticoid treatment. Table S3: primers used for real-time PCR analysis. Table S4: expression of top 10 abundant AMPs in nasal tissues from CRSwNP and healthy control identified by RNA sequencing. Figure S1: expression of CLU (A) and SLPI (B) in nasal tissues from uncinate process, inferior turbinate, and middle turbinate. CLU: clusterin; SLPI: secretory leukoprotease inhibitor. Figure S2: evaluation of sequential staining for AB/PAS and BPIFB2. AB/PAS staining and immunohistochemical staining for BPIFB2 were performed on serial sections of nasal tissues from ECRSwNP, nonECRSwNP, and controls. Bars = 50 μm. CRSwNP: chronic rhinosinusitis with nasal polyps; ECRSwNP: eosinophilic CRSwNP; BPIFB2: BPI fold-containing family B member 2. [file 1057186.f1.docx]
